# A Treatise on the Nervous System

**Published:** 1871-09

**Authors:** 


*A Treaties on the Nervous System.* By WILLIAM A. HAMMOND, M.D.

The medical profession will appreciate a systematic work upon Diseases of the Brain and Nerves, since enough is already known to fill a volume of near one thousand pages with well established truth. Many of these diseases have been studied anew within the past few years, and have thus been removed from the field of fancy and wild conjecture, and placed upon well defined basis of structural change. This work embraces a wide field, including all the diseases of the Brain and nervous system. The classification of disease and the method of presenting truth, merits the highest approval, he basis of the conclusion being given in such plain detail as to carry conviction to the mind of the attentive reader. The chapter on Insanity not proportionally more valuable than all others, is yet of great interest, partly on account of the attention which is now being paid to the study of mental diseases. The members of the profession who do not make insanity a special study, will find the recent views on this subject expressed in condensed form, so as to be adapted to the wants of general practitioners of medicine. All the other diseases of the Brain and Nerves, not associated with insanity, are also treated with equal ability and extent of research. It cannot fail of appreciation with the profession, and will take rank as one of our most valuable standard works on these subjects.

